# Clarifying the relationship between pulmonary langerhans cell histiocytosis and Alpha 1 antitrypsin deficiency

**DOI:** 10.1186/s13023-021-01720-9

**Published:** 2021-02-09

**Authors:** Cormac McCarthy, Emmanuelle Bugnet, Amira Benattia, Michael P. Keane, Benoit Vedie, Gwenaël Lorillon, Abdellatif Tazi

**Affiliations:** 1 Department of Respiratory Medicine, St. Vincent’s University Hospital, Dublin 4, Ireland; 2 School of Medicine, University College Dublin, Dublin 4, Ireland; 3 Hôpital Saint-Louis, Centre National de Référence des Histiocytoses, Service de Pneumologie, Assistance Publique-Hôpitaux de Paris, Paris, France; 4 Hôpital Européen Georges Pompidou, Service de Biochimie, Assistance Publique-Hôpitaux de Paris, Paris, France; 5 Université de Paris, INSERM U976, Institut de Recherche Saint-Louis, 75006 Paris, France; 6 Centre National de Référence des Histiocytoses, Service de Pneumologie, Hôpital Saint-Louis, 1 Avenue Claude Vellefaux, 75475 Paris Cedex 10, France

## Abstract

Pulmonary Langerhans cell histiocytosis (PLCH) is a rare, smoking related, progressive diffuse cystic lung disease that occurs primarily in smokers. The aim of this study was to determine if there was an increase in alpha-1 antitrypsin deficient alleles or phenotypes in a large series of PLCH patients and whether serum alpha-1 antitrypsin levels correlated with markers of disease severity. Fifty PLCH patients, 24 with a diffuse cystic lung pattern and 26 with a typical nodulo-cystic pattern on imaging were included. The mean alpha-1 antitrypsin levels were in normal range for both the population with diffuse cystic lung pattern population (1.39 g/L ± 0.37) and the nodulo-cystic pattern group (1.41 g/L ± 0.21). Deficiency alleles PiZ and PiS were 1% and 2% respectively in the entire study population of 50 patients, demonstrating no increased incidence of alpha-1 antitrypsin deficiency in PLCH. Alpha-1 antitrypsin levels showed no correlation with lung function parameters or extent of cystic lesions on lung computed tomography.

## Main text

Pulmonary Langerhans cell histiocytosis (PLCH) is a rare, smoking related, progressive diffuse cystic lung disease that occurs mainly in young smokers of both genders [1]. In adults, it is frequently the only manifestation of the disease, but may also be a part of systemic disease [1]. Findings on high resolution computed tomography (HRCT) varies from a nodulo-cystic pattern in recently diagnosed PLCH to a diffuse cystic lung disease pattern [2,3]. In the latter, the disease may be misinterpreted as emphysema, and given the smoking history the diagnosis may be difficult to delineate. Furthermore, in long standing PLCH patients superimposed emphysema can be observed. Approximately 50% of PLCH patients harbour somatic *BRAF*^V600^^E^ mutations in cells of the myeloid/monocyte lineage, and while the pathogenesis of PLCH is not entirely understood, it is likely related to smoking induced injury and induced dendritic cell dysfunction [4] and airway inflammation [5]. Langerhans cells accumulate in bronchiolar airways with lymphocytes, macrophages, and eosinophils, thought to result in tissue destruction and remodelling and resultant cyst formation. It has been proposed that metalloproteinases produced by inflammatory cells may contribute to destruction of extracellular matrix, including matrix metalloproteinases (MMPs) [6].

Alpha-1 antitrypsin (AAT) deficiency (AATD) is an autosomal codominant condition characterised by low circulating levels of AAT protein, the archetypal serine protease inhibitor. The most common variants associated with disease are the Z (Glu342Lys) and S (Glu264Val) mutations, caused by the substitution of glutamic acid for lysine or valine at positions 342 and 264 of the polypeptide [7,8]. AATD results in an increased risk of emphysema at a young age and the risk is increased with concomitant smoking [9]. In AATD there is an imbalance between proteases and antiproteases in the lung and there is abnormal inflammatory response and evidence of increased pulmonary inflammation [10,11]. Serine proteases may result in tissue injury and smoking may increase the amount present in the lung [12]. Consequently, it has been hypothesized that there may be a link between PLCH and AATD through a common pathway of proteolytic damage, further augmented by smoking [13]. In a small study conducted on 34 patients with PLCH there was an increased frequency of deficient AATD alleles noted, PiZ and PiS accounting for 5.88% and 2.94% of alleles [14]. To further clarify this proposed relationship, we sought to measure AAT levels and evaluate AAT phenotype in a large series of carefully classified PLCH patients, including both predominant diffuse cystic lung disease pattern and the nodulo-cystic pattern of PLCH.

## Methods

Fifty patients with a diagnosis of PLCH attending the National Reference Centre for Histiocytoses, Paris, France were included following informed consent (registry number: CNIL 909207; CCTIRS 09.191). All patients had diagnosis confirmed by either histology or based on typical HRCT findings either alone or in association with characteristic extrapulmonary involvement (e.g., lytic bone lesion, diabetes insipidus) following the exclusion of alternative diagnoses [3]. HRCT images were analysed at window levels appropriate for the pulmonary parenchyma (widths of -600-1600 HU) and the extent of the cystic lesions (including thick- and thin-walled cysts) was assessed as previously described [3]. Patients were classified into subgroups according to CT score values. For the whole lung, the maximum value of the cystic HRCT score was 24, with a total cystic lung score value greater than 12 denoting high (13–18) and very high (19–24) extent of cystic lung lesions respectively [3]. Lung volumes were evaluated by plethysmography, and forced expiratory volume in one second (FEV_1_) and forced vital capacity (FVC) were determined by flow curve volume. Diffusing capacity of carbon monoxide (D_LCO_) was measured using the single-breath method. Obstruction was defined as a ratio of FEV_1_ to FVC < 70%, lung restriction was defined as a total lung capacity (TLC) < 80% of the predicted value, and air trapping was defined as a ratio of residual volume (RV) to TLC > 120% of the predicted value. Serum/plasma concentration of AAT was determined by nephelometric assay (Beckman-Coulter, IMMAGE 800) with reference values of 1.0–2.7 g/L [15]. AAT phenotypes were assessed with Hydragel 18 A1AT Isofocusing kit on a Hydrasys System (Sebia) as previously described [16].

## Results

Twenty-four PLCH patients with a diffuse cystic lung pattern on HRCT were evaluated for A1AT, consisting of 15 current and 9 ex-smokers; 13 females and 11 males; and 9 patients with multisystem LCH. The median time between PLCH diagnosis and AAT serum measurement and phenotype analysis was 1 year [IQR, 0; 10]. PLCH diagnosis was histologically confirmed in 13 (54%) patients (lung n = 6, bone n = 2, skin n = 4, gut n = 1), the other 11 patients had either multisystem PLCH (n = 3) or had typical bizarre cysts. The median HRCT cyst score was 20 [IQR, 16.5; 22], 17 patients (71%) had airflow obstruction with a median FEV_1_% predicted of 58.5 [39.5–69.5]. Twenty-six additional PLCH patients (17 females, all current smokers) with a typical nodulo-cystic lung HRCT pattern were also evaluated for AAT levels and phenotype, with a median time between PLCH diagnosis and AAT assessment of 0 year [IQR, 0; 5] (Table 1). The mean AAT level in the diffuse cystic lung disease pattern population was 1.39 g/L (± 0.37) and 1.41 g/L (± 0.21) in the nodulo-cystic pattern group. One compound heterozygote PiSZ individual was identified with an AAT level of 0.54 g/L and one PiMS phenotype identified in the diffuse cystic cohort and all patients in the nodulo-cystic disease were PiMM. Deficiency alleles PiZ and PiS were 1% and 2% respectively in the entire cohort, while the incidence of PiZ and PiS in the French population are estimated at 7.6% and 1.3% [17], demonstrating that there is no increased incidence of AATD in PLCH. There was no correlation between AAT levels and FEV_1_ (R^2^ = 0.02135, p = 0.4957), FVC (R^2^ = 0.02930, p = 0.4239), D_LCO_ (R^2^ = 0.09381, p = 0.1552) or CT cyst scores (R^2^ = 0.1225, p = 0.093).

**Table 1:** Characteristics of PLCH patients included in the study

Characteristics	Diffuse cystic pattern n = 24	Nodulo-cystic pattern n = 26
Age, years, median, [IQR]	40 [31–46.75]	45 [34–47]
Female sex, n (%)	13 (54%)	17 (65%)
Histological diagnosis	13 (54%)	3 (12%)
Isolated PLCH, n (%)	15 (62.5%)	23 (88%)
MS LCH, n (%)	9 (37.5%)	3 (12%)
Time from PLCH diagnosis, years, median, [IQR]	1 [0–10]	0 [0–5]
Smoker, n (%)	15 (62.5%)	24 (92%)
Ex-smoker	9 (37.5%)	2 (8%)
Dyspnoea	18 (75%)	16 (62%)
NYHA II/III/IV	9/8/1	14/2
TLC, % of predicted	104 [92.75–123.75]	105 [95.25–123]
FVC, % of predicted	78 [69.25–87.25]	100 [84.25–114]
RV/TLC, % of predicted	148.5 [118.5–166.75]	111.5 [103–132.5]
FEV_1_, % of predicted	58.5 [39.5–69.5]	86.5 [76–100]
FEV_1_/FVC %	66 [48.25–72]	77 [69.25–80.75]
D_LCO_, % of predicted, *n* = *23*	37 [28–56]	52 [41–63]
Restriction* (%)	0	0
Obstruction (%)	17 (71%)	7 (27%)
Air trapping (%)	17 (71%)	10 (38%)
CT cyst score, median, [IQR]	20 [16.5–22]	N/A
Alpha-1 antitrypsin level (g/L) (mean, SD)	1.39 (± 0.37)	1.41 (± 0.21)
PiM allele frequency	94%	100%
PiS allele frequency	4%	0%
PiZ allele frequency	2%	0%

^*^Restriction was defined as TLC < 80% of predicted; obstruction as FEV_1_/FVC < 70% and air trapping as RV/TLC > 120% of predicted

PLCH, pulmonary Langerhans cell histiocytosis; AAT, alpha-1 antitrypsin; IQR, interquartile range; NYHA New York heart association, MS multisystem; TLC total lung capacity; FVC forced vital capacity; RV residual volume; FEV_1_ forced expiratory volume in 1 s; D_LCO_ diffusing capacity of carbon monoxide; N/A, not applicable

## Discussion

It has been previously hypothesized that there may be a link between PLCH and AATD. This is possibly due to shared mechanisms of proteolytic tissue damage. Previously it has been proposed that proteases produced by inflammatory cells in the lungs of PLCH may contribute to extracellular matrix destruction [6], and the primary mechanism of emphysema in AATD is the unopposed proteolytic damage of proteases due to lack of AAT to counteract this. Moreover, AATD is an inflammatory condition due to the reduced AAT capacity to modulate inflammatory cytokines, and similarly PLCH is an inflammatory condition [5]. Finally, it is evident that smoking affects protease activity and can alter the funciton of AAT ^12^. Hence, it was hypothesised that these two conditions may share a common pathogenic mechanism. Prior smaller studies have indicated a possible clinical link between the two rare diseases, however, our report is the biggest study to date, and we have demonstrated no increased rate of AATD or AAT deficient alleles in a large PLCH series. The prior hypothesis is not supported by this larger dataset, and moreover there was a lack of correlation between AAT level and pulmonary function or CT scores in PLCH. While indeed an imbalance between protease and antiprotease activity could contribute to cyst development in PLCH, whether this is metalloproteinase or serine protease driven remains unknown, it is apparent that there is no clinical link between these two rare lung diseases. To determine whether smoking related changes in antiprotease function plays a role remains unclear in PLCH. Further mechanistic studies may elucidate if there is any role of AAT protein either as an antiprotease or immune modulator in the pathogenesis of PLCH, but this seems unlikely to be a major player given the lack of correlation in this study.

## Data Availability

The data supporting the results reported in the current study are available from the corresponding author upon request.

## Abbreviations

AAT: Alpha-1 antitrypsin
AATD: Alpha-1 antitrypsin deficiency
D_LCO_: Diffusing capacity of carbon monoxide
FEV_1_: Forced expiratory volume in 1 s
FVC: Forced vital capacity
HRCT: High resolution computed tomography
IQR: Interquartile range
MMPs: Matrix metalloproteinases
PLCH: Pulmonary Langerhans cell histiocytosis
TLC: Total lung capacity
RV: Residual volume
